# Atomically precise semiconductor—graphene and *h*BN interfaces by Ge intercalation

**DOI:** 10.1038/srep17700

**Published:** 2015-12-07

**Authors:** N. I. Verbitskiy, A. V. Fedorov, G. Profeta, A. Stroppa, L. Petaccia, B. Senkovskiy, A. Nefedov, C. Wöll, D. Yu. Usachov, D. V. Vyalikh, L. V. Yashina, A. A. Eliseev, T. Pichler, A. Grüneis

**Affiliations:** 1 Faculty of Physics, University of Vienna, Strudlhofgasse 4, A-1090 Vienna, Austria; 2 II. Physikalisches Institut, Universität zu Köln, Zülpicher Straβe 77, D-50937 Cologne, Germany; 3 Department of Materials Science, Moscow State University, Leninskiye Gory 1/3, 119992, Moscow, Russia; 4 IFW Dresden, P.O. Box 270116, D-01171 Dresden, Germany; 5 St. Petersburg State University, 7/9 Universitetskaya nab, St. Petersburg, 199034, Russia; 6 Department of Physical and Chemical Sciences, University of L’Aquila, Via Vetoio 10, I-67100 L’Aquila, Italy; 7 CNR-SPIN, Via Vetoio 10, I-67100 L’Aquila, Italy; 8 Elettra Sincrotrone Trieste, Strada Statale 14 km 163.5, I-34149 Trieste, Italy; 9 Institute of Solid State Physics, Dresden University of Technology, Helmholtzstraße 10, D-01062 Dresden, Germany; 10 Institute of Functional Interfaces (IFG), Karlsruhe Institute of Technology (KIT), Hermann-von-Helmholtz-Platz 1, D-76344 Eggenstein-Leopoldshafen, Germany; 11 IKERBASQUE, Basque Foundation for Science, 48011 Bilbao, Spain; 12 Donostia International Physics Center (DIPC), Departamento de Fisica de Materiales and CFM-MPC UPV/EHU, 20080 San Sebastian, Spain; 13 JSC “Giredmet” SRC RF, Tolmachevky St. 5-1 B, 119017 Moscow, Russia; 14 Department of Chemistry, Moscow State University, Leninskiye Gory 1/3, 119992, Moscow, Russia

## Abstract

The full exploration of the potential, which graphene offers to nanoelectronics requires its integration into semiconductor technology. So far the real-world applications are limited by the ability to concomitantly achieve large single-crystalline domains on dielectrics and semiconductors and to tailor the interfaces between them. Here we show a new direct bottom-up method for the fabrication of high-quality atomically precise interfaces between 2D materials, like graphene and hexagonal boron nitride (*h*BN), and classical semiconductor via Ge intercalation. Using angle-resolved photoemission spectroscopy and complementary DFT modelling we observed for the first time that epitaxially grown graphene with the Ge monolayer underneath demonstrates Dirac Fermions unaffected by the substrate as well as an unperturbed electronic band structure of *h*BN. This approach provides the intrinsic relativistic 2D electron gas towards integration in semiconductor technology. Hence, these new interfaces are a promising path for the integration of graphene and *h*BN into state-of-the-art semiconductor technology.

In the recent years graphene attracted attention from researchers all over the world and was studied intensively because of its unique electronic properties showing promise for usage in next-generation electronics^1^. However, for commercial applications, implementation on a large scale into classical semiconductor technology is still missing^2^. Among other methods to grow graphene, chemical vapour deposition (CVD) on metallic substrates is known to achieve the highest quality of graphene sheets on a large scale^3^,^4^. Unfortunately, the electronic properties of epitaxially grown graphene are complicated by charge puddles, charge transfer, corrugation, and hybridization, modifying its unique relativistic two-dimensional (2D) electron gas^5^,^6^,^7^,^8^, while the post-growth transfer procedure inevitably leads to corrugation, contamination and breakage of graphene sheets^9^. Thus, many efforts were put to reduce the impact of the substrate on the electronic structure of graphene resulting in intercalation of metals^6^,^7^,^10^,^11^,^12^,^13^,^14^, semiconductors^15^,^16^,^17^,^18^, oxygen^19^,^20^,^21^ and hydrogen^22^,^23^ under epitaxially grown graphene. It has been shown that graphene can be grown on Ge by molecular beam epitaxy (MBE)^24^ or chemical vapor deposition (CVD)^25^ and that graphene on H-terminated Ge^4^ and on Ge oxide surface^26^ have superb electronic properties that can even exceed the mobilities of free-standing graphene membranes^26^. Up to now the best devices were obtained for graphene grown epitaxially on hexagonal boron nitride (*h*BN)^27^,^28^, i.e. achieving a novel van der Waals solid. Clearly, the electronic properties of such devices will be strongly influenced by the interface structure. So far these real-world applications are limited by the ability to concomitantly achieve large single-crystalline domains on dielectrics and semiconductors and to tailor the interfaces between the 2D material and the substrate. Even less is known about how to tailor interfaces between 2D materials, such as graphene and *h*BN, and classical semiconductors, such as Si and Ge, and especially about scalable growth of high-quality graphene and *h*BN sheets on semiconductors. Previous works on Ge intercalation were done for graphene on SiC for which the intercalated Ge is amorphous and no precise interface structures was reported^19^,^17^. In these works it was reported that Ge intercalation under graphene on SiC leads to ambipolar doping of ±0.3 eV^16^,^17^. Here we demonstrate a new bottom-up method for fabrication high-quality large-scale graphene Ge and *h*BN Ge heterostructures and a crucial pathway how to achieve an atomistic control on these interfaces while avoiding the post-growth transfer process. We experimentally prove the conditions how the Ge intercalation results in atomically precise interfaces between 2D material and atomically thin semiconductor layer and study the stability limits of these planar structures. Using state-of-art electronic structure characterization methods such as angle-resolved photoemission spectroscopy (ARPES) and complementary DFT modelling we observed that graphene with a Ge monolayer underneath demonstrates Dirac Fermions unaffected by the Ni substrate. Comprehensive studies also revealed an unperturbed electronic band structure of *h*BN after Ge intercalation. These results can have a huge implication for tailoring such interfaces and achieving devices based on relativistic 2D electron gas.

## Results

**Figure 1:**
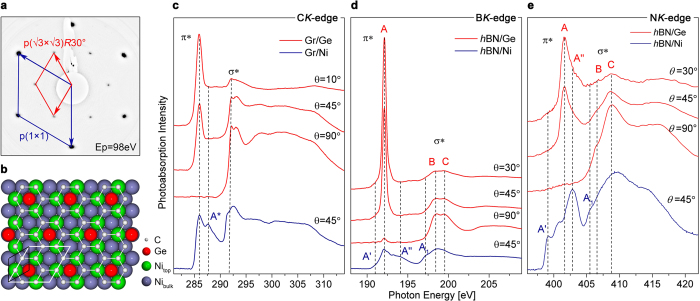
(**a**) LEED pattern of graphene after Ge intercalation showing 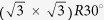 reconstruction. (**b**) Structural model (top view) corresponding to the LEED pattern with 6 C and 1 Ge atoms per unit cell. Ge atoms are shown in red, Ni atoms in purple, the topmost Ni atoms are shown in green. Angle dependent NEXAFS spectra measured before and after Ge intercalation for (**c**) graphene and (**d**,**e**) *h*BN. The corresponding adsorption edges are noticed in the Figures.

Epitaxially grown graphene and *h*BN on Ni(111) have a very well-known hexagonal *p*(1 × 1) low-energy electron diffraction (LEED) pattern^7^,^29^,^30^. Intercalation of Ge leads to a surface reconstruction which can be observed in LEED as a 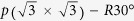 diffraction pattern as shown in Fig. 1(a). The LEED pattern of *h*BN after Ge intercalation is similar (Supplementary Figure S1). The diffraction spots are very sharp, showing the excellent crystallinity of the interface pointing towards a well-ordered Ge layer. The observed LEED pattern is consistent with a supercell with 6 C and 1 Ge atom per unit cell as shown in Fig. 1(b). This type of LEED pattern is also observed e.g. in LiC_6_ intercalated graphene^31^ and graphene/Co(0001), intercalated with silicon^32^. To explore further the structure and interaction with the substrate in Ge-intercalated graphene and *h*BN we employ near edge X-ray absorption fine structure (NEXAFS), X-ray photoemission (XPS), angle-resolved photoemission (ARPES) spectroscopies and *ab*-*initio* calculations.

NEXAFS spectroscopy allows one to probe unoccupied states and gives information about the hybridisation of electronic states. The angle dependence of NEXAFS K-edge spectra shows the orientation of the *π*-system with respect to the substrate and thus can be used to characterise the possible corrugation or flatness of the 2D film^33^,^34^. In Fig. 1(c–e) we show NEXAFS spectra of the graphene and the *h*BN *K*-edges before and after Ge intercalation. The absorption spectrum of graphene on Ni(111) is characterised by the presence of π^*^- and σ^*^-resonances and a feature A^*^ between them which is due to strong hybridisation between C2*p*_*z*_ and Ni3*d* orbitals of graphene and Ni substrate^35^. After Ge intercalation significant changes in the absorption spectra taken at *θ* = 45° can be observed–the A^*^ peak disappears, π^*^- and σ^*^-resonances become sharper and the lineshape of the spectrum becomes similar to quasi-free-standing graphene^36^. This points towards the decrease of hybridisation and decoupling of graphene from Ni substrate. The abscence of Ge3d states in the close vicinity of the Fermi level, as compared to Ni, leads to the abscence of hybridization between Ge and C, as compared to Ni. Another reason for such behaviour is the abscence of compounds or solubility at the intercalation temperature in Ge-C phase diagram, contrary to Ni-C phase diagram, where there are solid solution region and carbides. C*K*-edge NEXAFS spectra show a strong angular dependence with highest intensity of π^*^-resonance at grazing incidence and σ^*^-resonance at normal incidence, which shows that graphene on Ge is indeed very flat, as expected for quasi-free-standing graphene layer^36^. As can be seen in Fig. 1(d,e) both B and N*K*-edges of *h*BN acquired at *θ* = 45° change significantly after Ge intercalation. The spectral features *A*′, *A*″ and *A*_1_ significantly decrease. The main peak *A* of the π^*^-resonance becomes very intense, both π^*^- and σ^*^-resonances become more sharp and the spectra become similar to quasi-free-standing *h*BN^30^. These changes show the decrease of Ni3*d*–*h*BN*π* hybridisation as a result of Ge intercalation^37^,^38^. π^*^- and σ^*^-resonances show strong angular dependence with maximum intensity of π^*^ at grazing incidence and minimum at normal incidence, contrary to σ^*^, pointing towards a flat and decoupled *h*BN monolayer. Thus we can conclude that Ge intercalation leads to decoupling of graphene and *h*BN from metallic substrate and formation of quasi-free-standing graphene and *h*BN monolayers on Ge.

**Figure 2:**
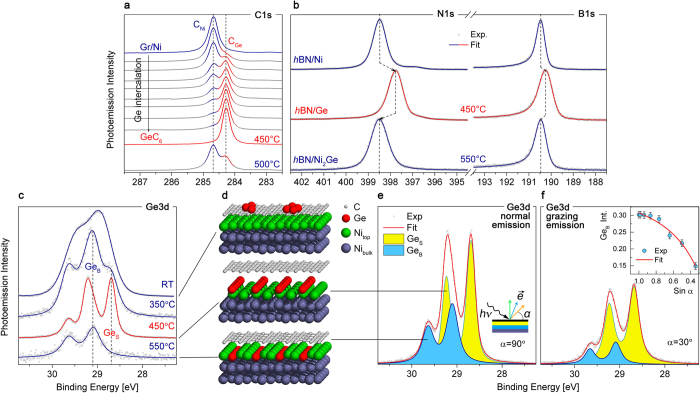
(**a**) C1*s*, (**b**) B1*s* and N1*s* core-level photoemission spectra before and after Ge intercalation. (**c**) Evolution of Ge3*d* core-level spectrum during annealing after deposition. (**d**) Structural model showing different steps during Ge intercalation. Ge3*d* XPS at (**e**) normal and (**f**) grazing photoelectron emission angles. The inset shows the fit of the relative intensity of *Ge*_*B*_ component with Beer-Lambert law.

To study the bonding environment and the stoichiometry in Ge-intercalated graphene and *h*BN we performed XPS measurements. In Fig. 2(a), the evolution of the C1*s* core level during Ge intercalation is depicted. It can be seen that after the first intercalation step the second component *C*_*Ge*_ appears in C1*s* line at lower binding energy. During further Ge intercalation the intensity of initial *C*_*Ni*_ component decreases while the intensity of *C*_*Ge*_ increases, pointing towards a liberation of graphene from the substrate. The fully intercalated sample is characterised by a single component *C*_*Ge*_ in the C1*s* line which is shifted by −0.41 eV as compared to graphene/Ni. The C1*s* binding energy of fully Ge-intercalated graphene is equal to that of quasi-free-standing graphene on Au(111)^39^. The same behaviour can be observed for the B1*s* and N1*s* core-level energies of *h*BN in Fig. 2(b). Here, Ge intercalation also leads to the shift of core-level photoemission peaks to a lower binding energy, indicative of the decoupling of *h*BN monolayer from the Ni substrate. N1*s* peak is shifted by −0.75 eV and B1*s* by −0.3 eV. This behaviour is similar to that of epitaxial *h*BN for Au intercalation^30^. According to the interface stoichiometry of GeC_6_ and GeB_3_N_3_, only 0.17 ML of Ge (with respect to graphene or *h*BN) are needed to form the interface. However, since part of Ge alloys with Ni, a larger amount of Ge is actually needed to form a complete GeC_6_ interface (according to quartz micro-balance). As we will show below, the Ge XPS spectrum also consists of a surface and a bulk component. Therefore, the ratio of the Ge surface component to the *C*_*Ge*_ component of the C1*s* spectrum is a measure of the interface stoichiometry. This stoichiometry, measured by XPS, rapidly approaches GeC_6_ in fully Ge-intercalated graphene, which is in a perfect agreement with LEED and our structural model. Further increasing the amount of deposited Ge leads to excessive surface coverage. This is observed as a reduction of the C1*s* photoemission intensity. To show the mechanism of Ge intercalation, we recorded the evolution of the Ge3*d* core-level spectrum during intercalation. As it can be seen in Fig. 2(c), as-deposited Ge has a broad and weakly resolved doublet corresponding to clusters adsorbed on the graphene surface [Fig. 2(d)]. During heating, the Ge atoms start to intercalate under the graphene (or the *h*BN) layer and the Ge3*d* line splits into two doublets – *Ge*_*B*_ and *Ge*_*S*_ (bulk and surface, correspondingly), separated by 0.4 eV. This is consistent with recent calculations, showing that a 2D layer of Ge is energetically preferred to small clusters^40^. Samples annealed to 450 °C represent the maximum intensity of the *Ge*_*S*_ component and correspond to fully Ge-intercalated graphene and *h*BN. Further annealing leads to the disappearance of the low-energy *Ge*_*S*_ component and is accompanied by shifting of the graphene and *h*BN core-levels back to the initial higher binding energy [see Fig. 2(a,b)].

To study the structure of the interface at fully Ge-intercalated graphene we measured the Ge3*d* core-level spectrum at different photoelectron emission angles to the sample surface and normalised it to the intensity of graphene’s (*h*BN’s) core-levels. As it can be seen in Fig. 2(e,f), the intensity of the Ge3*d* spectrum is lower at grazing emission, showing that after intercalation all Ge atoms are below the graphene or *h*BN layer. Also the relative intensity of *Ge*_*B*_ component is reduced with increasing emission angle which indicates that the corresponding Ge atoms are buried deeper under the surface. Thus we can conclude that the *Ge*_*S*_ doublet corresponds to intercalated Ge atoms in between graphene (*h*BN) and the Ni substrate and the *Ge*_*B*_ doublet correspond to Ni_2_Ge alloy, where Ge atoms are incorporated in topmost Ni layer. This conclusion is also supported by our simulations, the difference in Ge3*d* binding energy for intercalated and alloyed Ge atoms is 0.4 eV, which is in a perfect agreement with XPS data (see supplementary details for further information). The relative intensity of *Ge*_*S*_ component for different emission angles can be fitted with a Beer-Lambert law [see inset of Fig. 2(f)]. From this analysis we estimated the thickness of intercalated and alloyed Ge layers as 4.1 Å and 2.9 Å, correspondingly (A detailed description of the analysis procedure is given in Supplementary Note 1). The remarkably narrow spin-orbit split components in the Ge3*d* line show that Ge atoms after intercalation form a well defined structure, like it was observed for germanene^41^ and Ag_2_Ge surface alloy^42^. Interestingly, these samples are the first where the Ge doublet can be clearly observed, while previous attempts of Ge intercalation under graphene yielded only one Ge line and lacked a LEED pattern^16^,^17^. This information allows us to assume that Ge intercalation leads to formation of a well-ordered Ge layer in between graphene (*h*BN) [Fig. 2(d)] and the Ni(111) substrate at 450 °C and some amount of Ge atoms alloyed with Ni. Further annealing leads to alloying of all Ge intercalated with the Ni substrate and brings graphene (*h*BN) to the initial state with a higher binding energy [Fig. 2(a,b)]. Such transitions from the adsorbed state to a surface alloy have been observed before in Sn/Ni(111) system and resulted in the formation of an ordered surface alloy with the same structure^43^,^44^,^45^.

**Figure 3:**
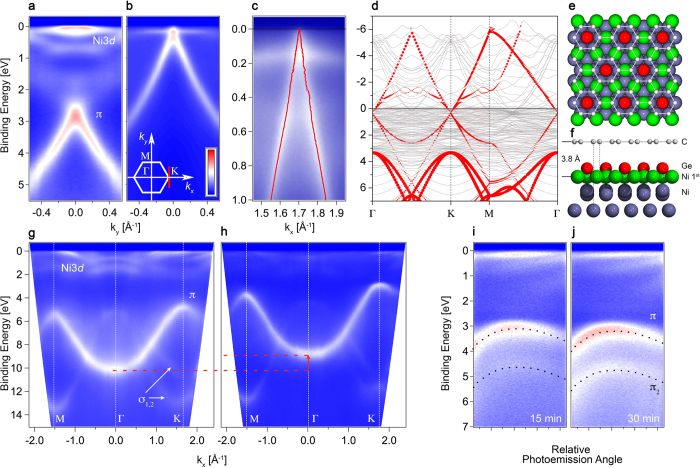
ARPES scans of (**a**) graphene/Ni(111) and (**b**) fully Ge-intercalated graphene, acquired perpendicular to the ΓKM high-symmetry direction in the vicinity of the K point. (**c**) High-resolution ARPES data of fully Ge-intercalated graphene acquired along ΓKM high-symmetry in the vicinity of K point. Red dotted lines denote ARPES intensity maxima. (**d**) Calculated band structure of graphene/1ML Ge/Ni(111). The size of red circles is proportional to the C*p*_*z*_ character of the eigenvalue. Structure model of graphene/1ML Ge/Ni(111) after geometry optimisation: (**e**) top view, (**f**) side view. C atoms are shown in gray, Ni atoms in purple, Ge in red, topmost Ni in green. ARPES scans of (**g**) *h*BN/Ni(111) and (**h**) *h*BN/Ge. ARPES images in the vicinity of the K point of *h*BN/Ge after annealing at elevated temperatures during (**i**) 15 and (**j**) 30 minutes.

To study the impact of Ge intercalation on the electronic band structure of graphene and *h*BN we performed ARPES measurements which directly probe the electron energy dispersion in solids. In Fig. 3(a,b) we show ARPES spectra in the vicinity of the K point of the graphene Brillouin zone before and after Ge intercalation. Figure 3(a) shows the well-known band structure of graphene on Ni(111). It can be characterised by a large shift of the graphene *π* band due to the strong hybridisation with Ni3*d* states, which brings the Dirac point to almost 3 eV binding energy. Ni3*d* states can be clearly observed at the Fermi level. Intercalation of Ge leads to drastic changes in the band dispersion of graphene. As we can see in Fig. 3(b), Ge intercalation restores the Dirac cone, shifting the Dirac point to the Fermi level, the intensity of Ni3*d* states decreases and the band structure of graphene becomes similar to that of quasi-free-standing graphene^6^. In Fig. 3(c) we show the high-resolution ARPES data measured at 20 K together with the momentum distribution curve (MDC) fit with two Lorentzian peak functions. It should be noted that because of matrix element effects in the photoemission cross section, the π^*^ graphene states are invisible with *p* polarization in the first Brillouin zone. Hence, we performed our photoemission studies in both, *p* and *s* polarization and then added them^39^,^46^. As it can be seen graphene on Ge is indeed quasi-free-standing – the Dirac point is at the Fermi level. This behaviour is different when compared to graphene on SiC intercalated with Ge^16^,^17^ and is attributed to the facts that (i) in the present case Ge is ordered and (ii) the substrate is different (Ni instead of SiC).

These experimental data are in good agreement with *ab*-*initio* calculations performed for Ge-intercalated graphene on Ni(111). We started from the optimised *top* − *fcc* geometry, which is known to be the most stable for graphene/Ni(111) theoretically^47^ and observed experimentally^5^. Then Ge was added in the right stoichiometry in between graphene and the topmost Ni layer in correspondence of the hollow site of graphene (lowest energy) and the structure was optimised. Atomic relaxation induced by the presence of Ge breaks the bonds between the surface Ni and C atoms. This produces a detaching of graphene from Ni(111) surface bringing it to about 3.8 Å, without any corrugation. The final geometry is characterised by a surface reconstruction of Ni which move away from the Ge adatom eventually sitting in correspondence of the middle of the C–C bond [Fig. 3(e,f)]. In the band structure linear Dirac bands belonging to the graphene lattice are restored and a Dirac point is present at about 0.3 eV from the Fermi level [Fig. 3(d)]. We summarised in Table 1 the calculated structural properties of the considered systems in terms of distances from substrate to graphene, corrugations and presence of the Dirac bands.

Figure 3(g,h) show the band dispersion of pristine *h*BN/Ni(111) and fully Ge-intercalated *h*BN. First, the intensity of Ni3*d* states at the Fermi level decreases significantly upon Ge intercalation. Second, the intercalation of Ge atoms leads to the shift of *π*-and *σ*-bands by 1.29 eV towards the Fermi level, bringing the *π* band at the K point to a binding energy of 2.9 eV. Such changes in the band structure point towards decoupling from Ni substrate and the formation of quasi-free-standing *h*BN monolayer^30^. We now discuss the effects of further annealing of Ge intercalated graphene and *h*BN on the electronic band structure. As it was mentioned in the discussion of the XPS data, further annealing of Ge intercalated graphene and *h*BN leads to alloying of all Ge atoms with the Ni substrate and diffusion of Ge into the bulk Ni. Figure 3(i,j) show the band dispersion of *h*BN/Ge in the vicinity of K point after annealing at elevated temperature (∼600 °C). It can be seen that such treatment leads to the appearance of a second band *π*_2_, whose intensity increases with the annealing time. Also the intensity of Ni3*d* states increases. This restores the initial electronic band structure of graphene and *h*BN with a higher binding energy.

**Figure 4:**
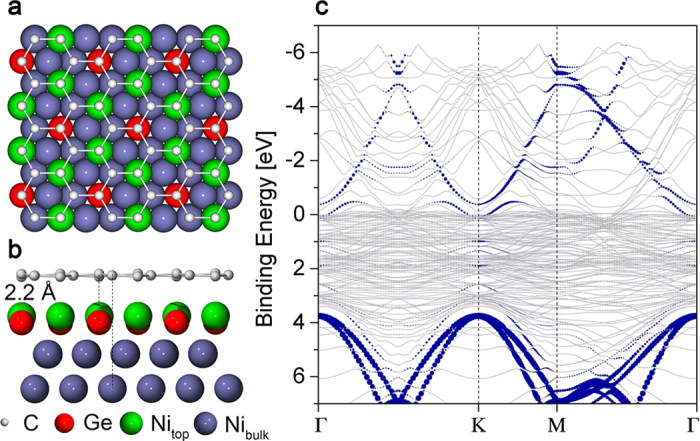
Structural model of graphene/Ni_2_Ge/Ni(111) in *top* – *fcc* geometry: (a) top and (b) side views. Ge atoms are shown in red, topmost Ni atoms in green and bulk Ni atoms in purple. (**c**) Calculated band structure of graphene/Ni_2_Ge/Ni(111). The size of coloured circles is proportional to the C*p*_*z*_ character of the eigenvalue.

Now we discuss a theoretical suggestion of an alternative structural model and provide reasons, based on our measurements, to exclude it. We performed ab-initio calculations for a structure with the same amount of Ge atoms (1 per unit cell) substituting Ni atoms in topmost layer, yielding the Ni_2_Ge alloy [Fig. 4(a,b)]. Incorporating of Ge in the topmost Ni layer produces a slight increase of the graphene–substrate distance up to 2.2 Å, as compared to graphene/Ni(111) (see Table 1), but the hybridisation between the Ni3*d* states and C*p*_*z*_ is still relevant and the Dirac cone is not restored [Fig. 4(c)]. Also graphene on the Ni_2_Ge layer shows a slight corrugation of 0.15 Å. This perfectly correlates with our experimental results and show that intercalation of Ge atoms leads to the formation of quasi-free-standing graphene and *h*BN, while formation of surface alloy with the same symmetry and stoichiometry does not lead to the decoupling of graphene (*h*BN) from the Ni substrate.

The calculated formation energy of graphene/Ni_2_Ge/Ni(111) is 1.4 eV lower than that of graphene/Ge/Ni(111) structure, which means that the formation of an alloy is more favorable and the driving force of Ge intercalation is the reduction of the total energy if Ge alloys with Ni. This explains the fact that at elevated temperatures the intercalated Ge layer overcomes the diffusion barrier and incorporates into the topmost Ni layer resulting in a Ni_2_Ge alloy.

## Conclusions

In this work we carried out a bottom-up approach to synthesize atomically precise graphene–Ge and *h*BN–Ge interfaces with GeC_6_ and GeB_3_N_3_ stoichiometry. Starting from epitaxial graphene/Ni(111) and *h*BN/Ni(111) we performed Ge intercalation which resulted in well-defined interfaces between the 2D layer and Ge. The 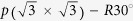 reconstruction was observed for both graphene–Ge and *h*BN–Ge systems after intercalation. Using XPS and LEED we have characterized the structure, stoichiometry and stability of these interfaces. Further NEXAFS and ARPES measurements and *ab*-*initio* calculations corroborated this structural assignment and showed that intercalation of Ge restores the graphene and *h*BN band structure making them quasi-free-standing. It was shown that intercalation leads to formation of atomically thin Ge layer, while further annealing leads to alloying of Ge with Ni and does not result in quasi-free-standing graphene or *h*BN. We have also shown that further annealing causes the reversal of the band structure changes since Ge diffuses inside the bulk Ni. Given the current interest in Ge nanostructures such as germanene and the promising transport properties of graphene on Ge, we believe that a well-defined interface with its electronic properties fully characterized can pave the way for future progress in this research direction.

## Methods

### Experimental details

Graphene/Ni(111) and *h*BN/Ni(111) samples were prepared *in situ* under ultra-high vacuum (UHV) conditions by chemical vapour deposition on Ni(111) single crystalline film from propylene (C_3_H_6_)^29^ and borazine (B_3_H_6_N_3_)^30^, respectively. Then Ge (99.9999 + at.%, MaTecK) was evaporated from a Ta crucible and intercalation was achieved by further annealing to 450 °C according to pyrometer. The total amount deposited was controlled by quartz microbalance and XPS (the scheme of the experimental setup is shown in the Supplementary Figure S2). The quality of the samples was controlled for each step by LEED. The XPS and NEXAFS measurements were carried out at HESGM^34^ and the German-Russian (RGBL)^48^ beamlines at the Helmholtz-Zentrum Berlin (Germany). XPS spectra were acquired at various emission angles with respect to the sample surface. C1*s* and Ge3*d* core-level spectra were acquired with *hv* = 330 eV excitation energy, N1*s* and B1*s* core-level spectra were measured with *hv* = 450 eV excitation energy. NEXAFS spectra were acquired in total electron yield (TEY) and partial electron yield (PEY) modes at different incidence angles *θ* and then normalised to the intensity of incident radiation. ARPES scans through the K point perpendicular to the ΓKM high-symmetry direction were acquired at UE112_PGM-1 beamline with the RGBL-2 station at Helmholtz-Zentrum Berlin (Germany). Spectra were acquired at a photon energy of 40 eV with the sample held at room temperature and a base pressure better than 5 × 10^−11^ mbar. High-resolution ARPES scans through the K point along the ΓKM high-symmetry direction were acquired at BaDElPh beamline^49^ at Elettra synchrotron in Trieste (Italy). ARPES spectra were acquired at a photon energy of 29 eV with the sample held at 20 K and a base pressure better than 5 × 10^−11^ mbar. The total angular and energy resolution was determined to 0.15° and 15 meV, respectively. ARPES measurements of *h*BN were performed using a photoelectron spectrometer equipped with a Scienta SES-200 hemispherical electron energy analyzer and a high flux He resonance lamp (Gammadata VUV-5010) in combination with a grating monochromator. Spectra were acquired at room temperature and a photon energy of 40.8 eV (He II*α*), with an angular resolution of 0.2° and a total energy resolution of 50 meV. Electron band dispersions were measured along the ΓKM direction of the Brillouin zone by varying the polar-emission angle.

### Computational details

The calculations were performed using the Vienna Ab-Initio Simulation Package (VASP)^50^,^51^ within the local density approximation (LDA)^52^. We used projected augmented-wave (PAW) pseudopotentials for all the atomic species involved with an energy cutoff up to 500 eV. Integration over the Brillouin Zone was performed considering an uniform 6 × 6 × 1 Monkhorst and Pack grid. The surfaces were simulated in the supercell approach with a vacuum space of 26 Å. Core-level shifts were calculated within the initial state approximation calculating the Kohn-Sham eigenvalues of the core states.

The formation energies (*E*_*f*_) were calculated as:


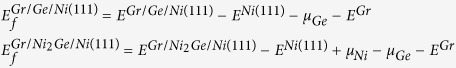


where *E*^*X*^ is the Kohn-Sham total energy of the *X* system. The chemical potentials of Ge and Ni (*μ*_*Ge*_ and *μ*_*Ni*_, respectively) where considered equal to the total energy/atom of their stable bulk phases.

## Additional Information

**How to cite this article**: Verbitskiy, N. I. *et al.* Atomically precise semiconductor—graphene and *h*BN interfaces by Ge intercalation. *Sci. Rep.* **5**, 17700; doi: 10.1038/srep17700 (2015).

## Supplementary Material

Supplementary Information

## Figures and Tables

**Table 1:** Calculated structural properties of graphene/Ni(111), graphene/Ge/Ni(111) and graphene/Ni_2_Ge in terms of distances from substrate to graphene, corrugations and presence of the Dirac bands.

System	Distance [Å]	Corrugation [Å]	Dirac Cone
graphene/Ni(111)	2.0	0.02	NO
graphene/Ge/Ni(111)	3.8	0	YES
graphene/Ni_2_Ge(111)	2.2	0.15	NO
