# Supplementary material for: Atomically precise semiconductor—graphene and hBN interfaces by Ge intercalation
Source: Sci Rep. 2015 Dec 7;5:17700. doi: 10.1038/srep17700 (PMC4671056; doi:10.1038/srep17700)
Supplement: Supplementary Information [file srep17700-s1.doc]

**Supplementary Information**

**Atomically precise semiconductor – graphene and *h*BN interfaces by Ge intercalation**

N.I. Verbitskiy,^†,‡,¶^ A.V. Fedorov,^‡,§,∥^ G. Profeta,^⊥,#^ A. Stroppa,^#^ L. Petaccia,@ B. Senkovskiy,^△,∇,‡^ A. Nefedov,^††^ C. Wöll,^††^ D.Yu. Usachov,^∥^ D.V. Vyalikh,^△,∥^ L.V. Yashina,^‡‡,¶¶^ A.A. Eliseev,^¶^ T. Pichler,^†^ and A. Grüneis^∗,‡^

*^†^Faculty of Physics, University of Vienna, Strudlhofgasse 4, A-1090 Vienna, Austria*

*^‡^Institute of Physics II, University of Cologne, Zülpicher Straße 77, D-50937 Cologne, Germany*

*^¶^Department of Materials Science, Moscow State University, Leninskiye Gory 1/3, 119992, Moscow, Russia*

*^§^IFW Dresden, P.O. Box 270116, D-01171 Dresden, Germany*

*^∥^St. Petersburg State University, St. Petersburg, 198504, Russia*

*^⊥^Department of Physical and Chemical Sciences, University of L’Aquila, Via Vetoio 10, I-67100 Coppito, Italy*

*^#^CNR-SPIN, Via Vetoio 10, I-67100 Coppito, Italy*

*@Elettra Sincrotrone Trieste, Strada Statale 14 km 163.5, I-34149 Trieste, Italy*

*^△^Institute of Solid State Physics, Dresden University of Technology, Helmholtzstraße 10, D-01062 Dresden, Germany*

*^∇^St. Petersburg State University, St. Petersburg, 198504, Russia*

*^††^Institute of Functional Interfaces (IFG), Karlsruhe Institute of Technology (KIT), Hermann-von-Helmholtz-Platz 1, D-76344 Eggenstein-Leopoldshafen, Germany*

*^‡‡^JSC “Giredmet” SRC RF, Tolmachevky St. 5-1 B, 119017 Moscow, Russia*

*^¶¶^Department of Chemistry, Moscow State University, Leninskiye Gory 1/3, 119992, Moscow, Russia*

*Correspondence to nikolay.verbitskiy@univie.ac.at

**Supplementary Figures**


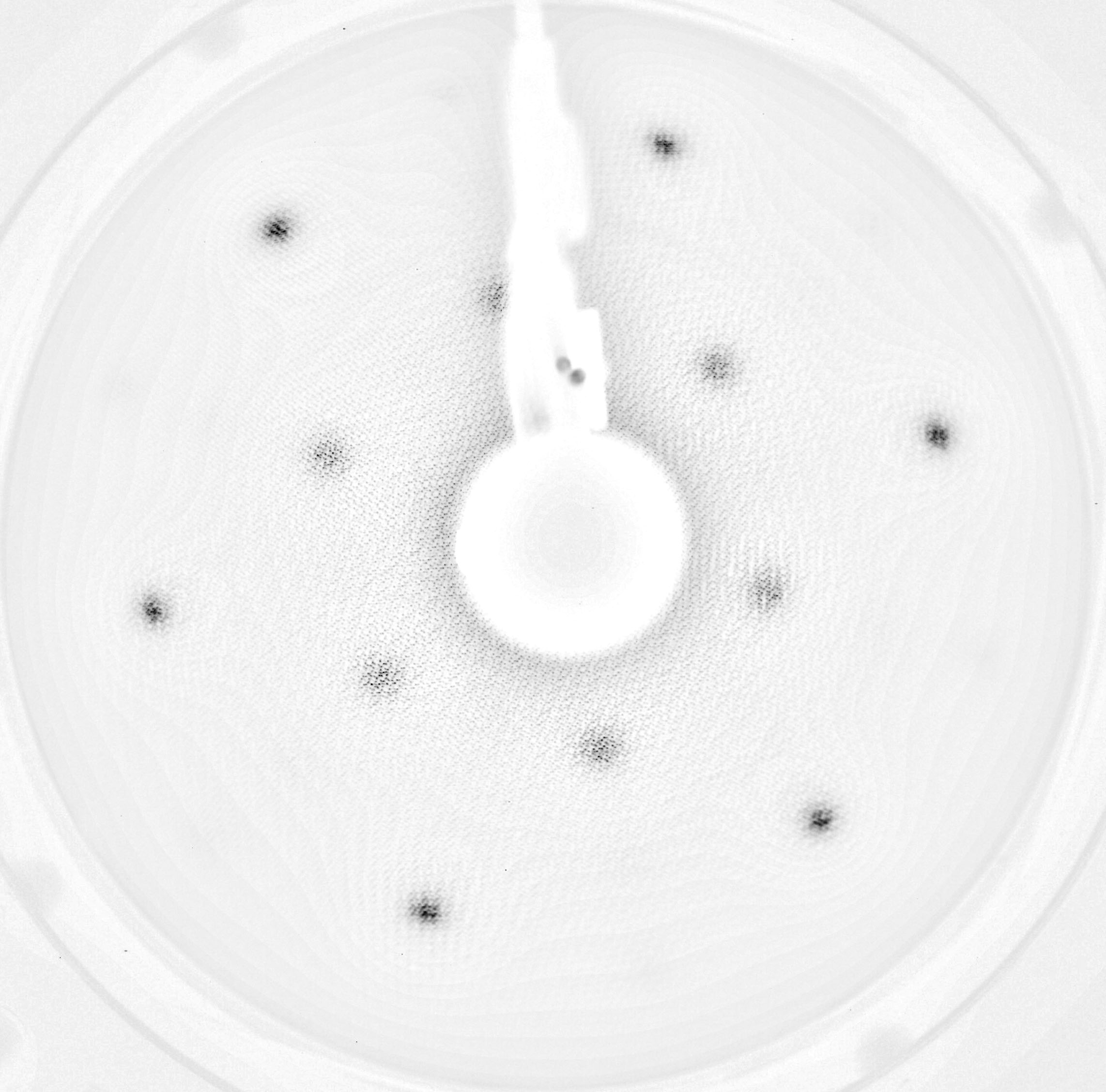


**Supplementary Figure S1:** Low-energy electron diffraction (LEED) pattern of *h*BN/Ni after Ge intercalation, showing (√3×√3)–*R30◦* surface reconstruction. (Ep=103 eV)

**
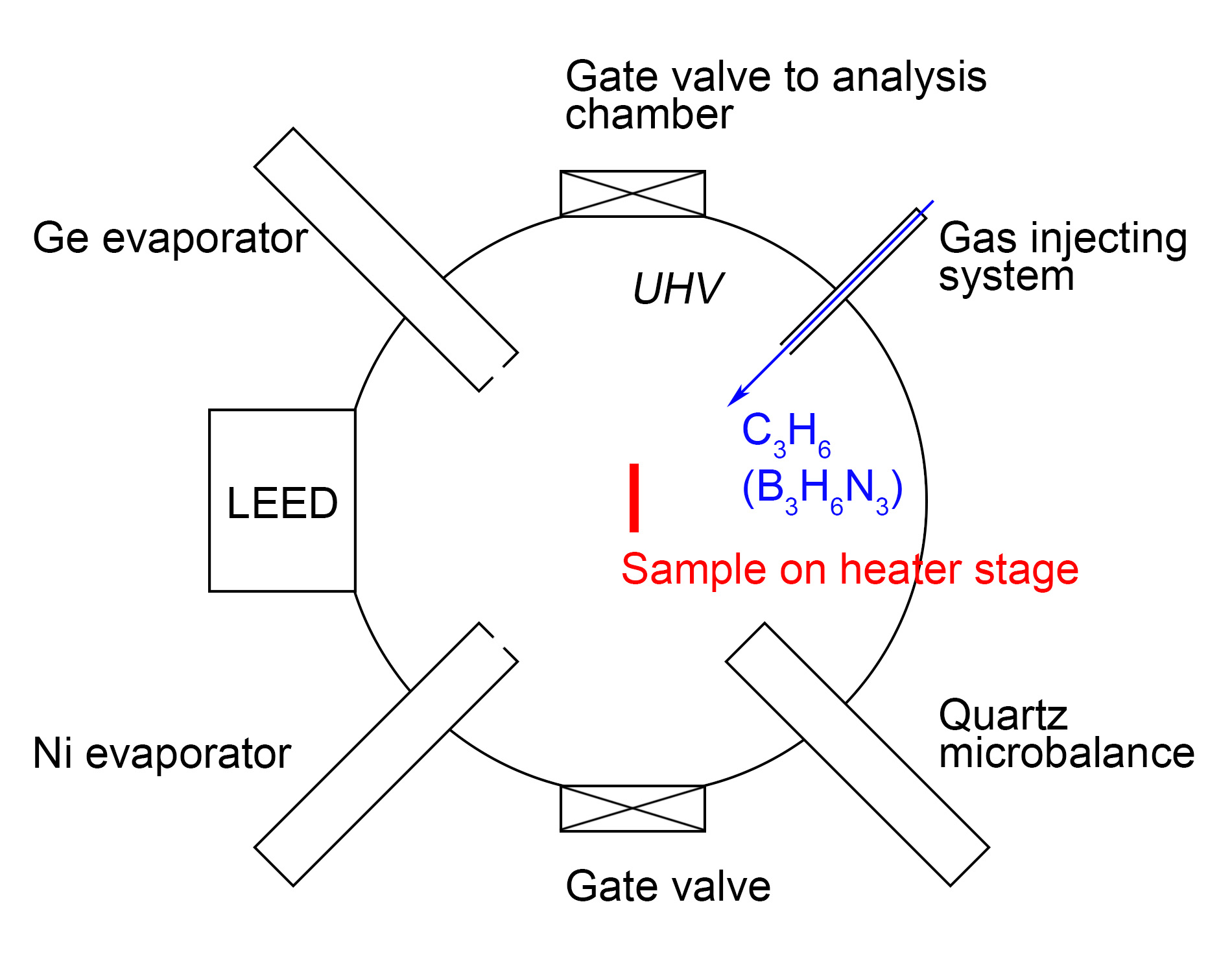
**

**Supplementary Figure S2:** Schematic CVD setup used for preparation and analysis of graphene/Ge/Ni and *h*BN/Ge/Ni interfaces

**Supplementary Note 1:**

The stoichiometry in the graphene/Ge/Ni and *h*BN/Ge/Ni samples was determined using X-ray photoelectron spectroscopy (XPS). The core-levels spectra of the 2D films (C1*s* in case of graphene, B1*s* and N1*s* in case of *h*BN) were measured together with the Ge3*d* line. Spectra were fitted with pseudo-Voigt profiles and the Shirley background. Spectra of the 2D film and intercalated Ge layer were measured with the same excitation energy and pass energy. After normalization to the photoemission cross section the ratio of the peaks resulted in the corresponding atomic ratio in the sample.^S1^ During this normalization we also took into account the scattering of photoelectrons on overlying layers. ^S2,S3^

XPS is a powerful method allowing studying of not only the bonding environment and stoichiometry in the samples but also depth profiling, by varying the emission angle of the photoelectrons. We used this technique, called angle-dependent XPS to study the relative depth of Ge layers and their thickness after intercalation. The intensity of electrons, emitted at an angle *θ* to the surface normal is given by the Beer-Lambert relationship:

*I = I_0_ exp(-d / λcosθ),*

where *I* is the intensity of the photoelectrons, *I_0_* is the intensity from an infinitely thick, uniform substrate, *θ* is the photoelectrons emission angle, *λ* is the inelastic mean free path and *d* is the thickness of layer from which the photoemission occurs.

After Ge intercalation under graphene and *h*BN and successful decoupling of 2D layers from Ni substrate (according to NEXAFS data) we assumed that graphene (*h*BN) layer is the top layer in the sample. Then C1*s* and Ge3*d* core-levels were measured using the same excitation energy, analyzer pass energy and with the same number of scans. These measurements were performed at different emission angles to the analyzer. The XPS spectra at different emission angles were normalized to the C1s intensity. After this normalization the lower intensity of Ge3*d* line at grazing emission means that Ge layer is under graphene and a higher Ge3*d* intensity means that Ge is on top. Moreover, the use of Beer-Lambert relationship, described above, allows estimating the thickness of layer from which the photoemission occurs^S1^.

**Supplementary References:**

^S1^ Watts, J.F. & Wolstenholme, J. in *An introduction to surface analysis by XPS and AES*, Ch. 4, 79-110 (Wiley 2003)

^S2^ Opila, L. R. & Eng, J. Jr, Thin films and interfaces in microelectronics: composition and chemistry as function of depth. *Progr. Surf. Sci*. **69(4)**, 125-163 (2002)

^S3^ Jablonski, A. & Zemek, J. Overlayer thickness determination by XPS using the multiline approach. *Surf. Interface Anal.* **41(3)**, 193-204 (2009)
